# Effect of different extraction methods on antioxidant properties and encapsulation efficiency of anthocyanin of pomegranate peel

**DOI:** 10.1002/fsn3.3362

**Published:** 2023-04-21

**Authors:** Niloofar Zahed, Reza Esmaeilzadeh Kenari, Reza Farahmandfar

**Affiliations:** ^1^ Department of Food Science and Technology Sari Agricultural Sciences and Natural Resources University Sari Iran

**Keywords:** FT‐IR, microwave, phenolic compounds, solvent, ultrasound

## Abstract

This study aimed to measure the efficiency, total anthocyanin content (TAC), and total phenol content (TPC) of pomegranate peel powder (PPP) extract from different extractions. Also, the characteristics of the nanoencapsulated extracts with maltodextrin (MD)/*Lepidium perfoliatum* (Qodume Shahri) seed gum were investigated. The highest and lowest extraction efficiency was related to solvent ethanol–water extraction (SEWE) (76.35%) and solvent ethanol extraction (SEE) (25.73%), respectively. Extracts obtained from microwave extraction (ME) and ultrasound extraction (UE) methods had the highest and lowest values of TAC (4.00–0.35) (mg C3G/g PPP) and TPC (702.13–232.58) (mg GAE/100 g sample), respectively. Peak 3213 in FT‐IR indicates the O–H bond, which showed the highest content of phenolic compounds in the extract obtained from ME compared with SEE, SEWE, and UE. The nanoencapsulated extracts from SEE, SEE, and UE had the lowest particle size of peak 1, particle distribution in peak 1, and average particle size distribution compared with other extractions, respectively. The highest encapsulation efficiency of anthocyanin (EEA) and encapsulation efficiency of phenol (EEP) were related to UE (96.15%) and SEWE (86.57%), respectively. The EEP and EEA of SEE were not significantly different from ME and SEWE, respectively. On the other hand, the type and amount of extractive compounds in the extract have a great impact on the efficiency of nanoencapsulation and the average size distribution of nanoencapsulated particles. As a result, PPP extract is rich in antioxidant compounds, which can be determined by carefully examining the appropriate method of extraction and preservation of the extracted compounds.

## Graphical abstract

Nanoencapsulation of anthocyanin compounds improves their antioxidant properties and leads to their greater stability.

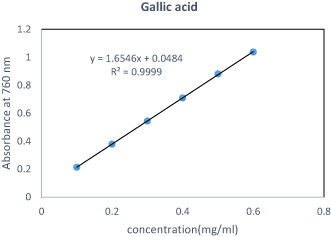

## 1 INTRODUCTION

Recently, due to many concerns about the carcinogenicity of synthetic antioxidants, the use of natural antioxidants has been considered. Scientific information on the antioxidant properties of various plants, especially those less commonly used in food and medicine, is still lacking. Therefore, it is practical to evaluate such properties, especially to find new sources of natural antioxidants (Arabshahi‐Delouee & Urooj, 2007). On the other hand, the interest in developing food colors from natural sources as an alternative to artificial colors has increased due to legal measures and consumer concerns. Anthocyanins belong to a broad group of phenolic compounds commonly called flavonoids. Anthocyanins and phenolic compounds can activate antioxidants by donating hydrogen to radicals and preventing the production of more oxidation products (Assous et al., 2014). Inexpensive resources and food industry remnants are the focus of more attention. Fruit peels are valuable wastes that can be obtained from industrial and domestic use. One of the natural sources of food coloring is anthocyanin extract from pomegranate peel (PP).

Pomegranate fruit (*Punica granatum* L.) is abundantly produced in Iran. This fruit can be consumed fresh, as a juice, drink, or in other food products (Abid et al., 2017). Health effects of pomegranate fruit: In addition to the edible part, it also includes non‐edible parts (especially the peel, which contains biologically active compounds). PP has more phenolic compounds than meat and seeds. PP can be a good source of high‐value antioxidants (Essa & Mohamed, 2018; Shaban et al., 2013).

The extraction process is a critical step and plays an important role in determining the optimal amount of bioactive compounds such as anthocyanins. Parameters affecting the yield of bioactive compounds from plant sources include the following: plant matrix, type of solvent, temperature, pressure, time, amount of solvent, and liquid/solid ratio (Azmir et al., 2013; Ilaiyaraja et al., 2015). Anthocyanins are usually extracted with acidic solvents under mild conditions (Puértolas et al., 2013). This solvent system destroys cell walls and membranes while dissolving, and stabilizing anthocyanins (Navas et al., 2012). The use of ultrasound in plant extraction has the advantages of increasing mass transfer, better solvent penetration, less dependence on the solvent used, extraction at low temperatures, faster extraction speed, and higher crop yields (Rana & Meena, 2017). Researchers prefer to use the ME method because it is cheaper, requires less time, has higher extraction efficiency, and also has a better yield product (Maran et al., 2013).

Encapsulation is used in the food industry to control the release of perfumes, flavorings, bioactive substances, and foods containing probiotics (Kuang et al., 2010). Encapsulation is a method of coating materials (such as natural dyes) in the form of micro and nanoparticles (Khazaei et al., 2014). The components of the wall, in addition to being natural, must have maximum protection of the active materials against environmental conditions, keep the active materials inside the capsule structure during processing or storage in different conditions, and have good rheological properties (Elsebaie & Essa, 2018). The most widely used for coating in food are polysaccharides, which contain starch and their derivatives – MD, as well as gums (Wandrey et al., 2010). The use of indigenous gums such as LPSG has been considered because of their cheapness. So far, no research has been done on the nanoencapsulating of anthocyanin extract of PPP with LPSG. The purpose of this work is to study the effect of different extraction methods on antioxidant properties and the combination of LPSG and MD on the encapsulation efficiency of anthocyanin extracts of PP. In this study, solvent, microwave, and ultrasound were used to extract phenolic compounds and anthocyanins from PPP. It was then encapsulated with MD and LPSG. The effects of different extraction methods on the content of anthocyanins, phenolic compounds, and nanoencapsulation properties of PPP extract were evaluated.

## 2 MATERIALS AND METHODS

### 2.1 Materials

First, 5 kg of Saveh red pomegranate fruit was first prepared. The PP was removed from the fruit and then dried for 72 h at room temperature, away from sunlight. Then, it was crushed, and a sieve (mesh 18) was used to prepare the powder. It kept in the refrigerator until use. Qadomeh Shahri seeds were purchased from Attarak Company. All the chemical materials were purchased from Sigma‐Aldrich.

### 2.2 Methods

#### 2.2.1 Solvent extraction

Extraction was performed by the method of Fuleki and Francis (1968) with some modifications; 20 g of PPP was combined with 150 mL of acidified ethanol (0.01% citric acid) and a mixture of acidified ethanol and water (1:1) for 5 min with an electric mixer soaked at maximum speed (Folki & Francis, 1968).

#### 2.2.2 Microwave extraction

Extraction was performed by the method of Duan et al. (2015). A quantity of 1 g of PPP was mixed with 20 mL of 96% ethanol and 1% hydrochloride. After mixing, the sample was placed in a microwave (45°C: 360 w: 7 min; Duan et al., 2015).

#### 2.2.3 Ultrasound extraction

Pomegranate peel powder was extracted by bath ultrasound according to the method of Chranioti et al. (2015). About 1 g of PPP was mixed with distilled water (20 mL) and placed in a bath ultrasound (Elmasonic S) (30°C: 37 kHz: 60 min; Chranioti et al., 2015).

After each extraction, the extracts were filtered with Whatman 42 filter paper by the Buchner funnel. The extracts were centrifuged by refrigerated centrifugation (5 min: 10°C: 7168 *g*). Then the supernatant was collected and placed in an oven (45°C) to evaporate the solvent. The final solution of each extraction was kept at −18°C (Assous et al., 2014).

#### 2.2.4 Extraction efficiency

Extraction efficiency was calculated using the following equation:

$$\text{Extraction efficiency}=\frac{\text{Weight of the extract}}{\mathrm{PPP}\;\text{weight}\;}\times 100$$

#### 2.2.5 Total anthocyanin content

Total anthocyanin content was determined by the pH differential method, as described by Kırca and Cemeroğlu (2003). About 1 mL of anthocyanin extract of PPP was mixed with 9 mL of distilled water and then mixed with potassium chloride buffer solution at pH = 1 and sodium acetate at pH = 4.5. The absorption was recorded at 520 and 700 nm using UV–VIS spectrophotometer after 20 min (Model 1502) (Kırca & Cemeroğlu, 2003).

$$A=\left(A_{520\;\mathrm{nm}}–A_{700\;\mathrm{nm}}\right)\;{\mathrm{pH}}_{1.0}-\left(A_{520\;\mathrm{nm}}–A_{700\;\mathrm{nm}}\right)\;{\mathrm{pH}}_{4.5}$$

$$\mathrm{TAC}=\frac{A\times \mathrm{MW}\times \mathrm{DF}\times 1000\times V}{\varepsilon \times L\times M\;}$$

*A* = absorption, MW = molecular weight of cyanidin‐3 glucoside (449.2 g/mol), DF = dilution factor, *V* = volume of extraction (mL), *ε* = molar absorption coefficient of cyanidin‐3 glucoside (29,600 L/mol cm), *L* = cell length (cm), *M* = Pomegranate peel powder weight (*g*).

#### 2.2.6 Total phenol content

Total phenol content (TPC) was determined by the Folin–Ciocalteu method (Singh et al., 2002). First, 1 mL of the extract at a concentration of 1 mg/mL was mixed with 5 mL of 10‐Folin–Ciocalteu reagent. A volume of 4 mL of 7.5% sodium carbonate was added. It was placed at 27°C for 30 min, and the absorbance was measured at 760 nm using a UV–VIS spectrophotometer. The results were equivalent to mg of GAE/100 g of sample. Gallic acid was used as a standard to make a calibration curve (Kuspradini et al., 2016).

#### 2.2.7 Extraction of the gum of Qodume Shahri seeds

First, the impurity present in the seed of Qodume Shahri was separated, and then its gum was extracted (seed/water = 1/30 at 48°C and pH = 8) according to the method of Koocheki et al. (2009). The pH of distilled water was adjusted with NaOH solution. Then it was placed in a 48°C water bath. After the bath reached the desired temperature, seeds were added to it. It was stirred continuously (2 h) to increase the absorption of water by the seeds. A juicer was used to extract gum. The resulting extract was dried (70°C) in an oven. Finally, after grinding and sieving (mesh 40), the gum powder was stored in closed containers in the refrigerator for use in nanoencapsulation (Razavi et al., 2011).

#### 2.2.8 Pomegranate peel extract nanoencapsulating

A combination of 0.5 g of LPSG and 4.5 g of MD was used to nanoencapsulate the anthocyanin extract. MD was mixed with LPSG powder then distilled water was gently added to the solids to 15%. Then anthocyanin extract was added to the coating materials in a ratio of 1:2 (w/w) and homogenized using ultratorax (21952 *g*: 5 min). To further reduce the particle size, a prop ultrasound was used for 6 min (10 s per cycle and 2 s rest). It was frozen for 24 h (−18°C), then it was dried by a freeze‐dryer (48 h) (Vaco 5, Zirbus) (Carneiro et al., 2013).

#### 2.2.9 Particle size

Nanoencapsulated compound particle size was measured by Scatroscope 1 (manufactured by Qudix; Sadeghian et al., 2013).

#### 2.2.10 FT‐IR

FT‐IR (Cary 630, Agilent) with a wavelength of 650–4000 cm^−1^ was used to identify the nanoencapsulated compounds (Jamshidi et al., 2020).

#### 2.2.11 Encapsulation efficiency of anthocyanin (EEA)

About 2 mL of distilled water was added to 200 mg of samples. Then 18 mL of ethanol was added to and filtered with Whatman 42 paper. Total anthocyanin (TA) was calculated using the following equation (Lee et al., 2005):

$A=\left(A_{520\;\mathrm{nm}}–A_{700\;\mathrm{nm}}\right)\;{\mathrm{pH}}_{1.0}-\left(A_{520\;\mathrm{nm}}–A_{700\;\mathrm{nm}}\right)\;{\mathrm{pH}}_{4.5}$

$$\mathrm{TA}=\frac{A\times \mathrm{MW}\times \mathrm{DF}\times 1000\times V}{\varepsilon \times L\times M\;}$$

To measure the surface anthocyanin (SA), 100 mg of the nanoencapsulated extract was added to 10 mL ethanol. Then it was centrifuged (15°C: 1792 *g*: 5 min). The supernatant was removed. SA was calculated through differential pH. EEA was calculated from the following formula (Barbosa et al., 2005):

$$\mathrm{EEA}\%=\frac{\mathrm{TA}-\mathrm{SA}}{\mathrm{TA}}\times 100$$

#### 2.2.12 Encapsulation efficiency of phenol

Encapsulation efficiency of phenol (EEP) was determined according to the method of Kaderides et al. (2015). To measure the total phenol (TP), 5 mg of the sample was added to 5 mL of distilled water. It was then mixed with a magnetic stirrer for 5 min. It was then filtered with Whatman 42 paper. To measure the surface phenolic compounds (SP), 5 mg of the sample was added to 5 mL of distilled water. The surface of the mixture was quickly removed, and the Folin–Ciocalteu method was used to measure TP and SP (Singh et al., 2002). EEP was calculated using the following equation:

$$\mathrm{EEP}\%=\frac{\mathrm{TP}-\mathrm{SP}}{\mathrm{TP}}\times 100$$

### 2.3 Statistical analysis

Data in this study were analyzed using SAS software version 9.4 by one‐way analysis of variance (ANOVA) and Duncan test (*p*_Value_ ≤ .05). The results were expressed as mean with standard deviation. Experiments were performed in three replications.

## 3 RESULTS AND DISCUSSION

### 3.1 Extraction efficiency

The different extraction conditions are given in Table 1. In Table 2, there is a significant difference in the level of confidence above 95% of the yields obtained from different extracts of PPP. The highest rate of extraction related to SEWE was 76.35 ± 1.44 and the lowest rate of extraction related to SEE was 25.73 ± 1.67. Researchers found in a study that the solvent most commonly used to extract polar flavonoids (anthocyanins) is a mixture of water and organic solvents (Chen et al., 2008). The researchers obtained the best extraction efficiency from the combination of ethanol solvent with different concentrations (35%–90%) and water (Chaves et al., 2020). Researchers reported the highest yield with water–ethanol (50:50) and the lowest yield with water (Malviya et al., 2014). For this reason, some authors recommend that despite its safety and low cost, water is not suitable for the extraction of phenolic compounds. Recently, researchers have estimated that mixing ethanol: acidic water with acetic acid increases extraction performance (Masci et al., 2016). Inequality between extraction efficiencies can be explained by the difference between the solubility of phenols between solvents (Li et al., 2006).

**TABLE 1:** Conditions for different extractions of pomegranate peel powder of different extractions.

Sample	Solvent	Temperature (°C)	Time (min)	Solid/Solvent	Situation device
SEE	Ethanol/Citric acid 0.01%	27	5	1:7.5	–
SEWE	Ethanol/Water/Citric acid 0.01%	27	5	1:7.5	–
ME	Ethanol/HCl 1%	45	7	1:20	Power 360 w
UE	Water	30	60	1:20	Frequency 37 kHz

Abbreviations: ME, microwave extraction; SEE, solvent ethanol extraction; SEWE, solvent ethanol–water extraction; UE, ultrasound extraction.

**TABLE 2:** Efficiency extraction, TAC and TPC of different extractions.

Sample	Efficiency extraction	TAC (mg C3G/g PPP)	TPC (mg GAE/100 g E)
SEE	25.73 ± 1.67^d^	2.05 ± 0.12^c^	589.31 ± 4.24^b^
SEWE	76.35 ± 1.44^a^	3.14 ± 0.03^b^	418.70 ± 9.81^c^
ME	46.05 ± 1.22^b^	4.00 ± 0.59^a^	702.13 ± 10.93^a^
UE	40.23 ± 1.85^c^	0.35 ± 0.13^d^	232.58 ± 4.63^d^

*Note*: Different letters in a column indicate significant differences (*p* < .05).

Abbreviations: C3G, cyanidin‐3 glucoside; E, extract; GAE, gallic acid equivalent; ME, microwave extraction; PPP, pomegranate peel powder; SEWE, solvent ethanol‐water extraction; SEE, solvent ethanol extraction; TAC, total anthocyanin content; TPC, total phenol content; UE, ultrasound extraction.

### 3.2 Total anthocyanin content

Table 2 shows the value of TAC in different extracts. The highest amount of TAC measured by differential pH by spectrophotometric colorimetry related to ME extract is equivalent to 4.00 mg/g PPP, and the lowest amount of anthocyanin composition with a large difference from other methods for UE extract is 0.35 mg/g PPP. Some studies have reported that extraction solvents can be acidified to protect sensitive flavonoids (anthocyanins) from oxidative degradation (Dzah, 2014), which produces hydrogen ions (H^+^) that are free radicals which may be produced during UE (Dzah et al., 2020). This study actually explains the reason for the low amount of anthocyanin extracted by ultrasound in the current study. In a study by Rababah et al. (2010), the amount of anthocyanin in PP was reported to be 1.7–1.3 mg/g dry weight, which is consistent with the results of this study.

### 3.3 Total phenol content

The TPC is shown in Table 2. TPC for ME was equal to 702.13 mg GAE/100 g of sample, which was higher than SEE, SEWE, and UE, respectively. The results also showed that ethanol as a solvent is more effective than the combination of water–ethanol and water in extracting phenolic compounds from PPP. A study showed that increasing the ratio of solvent to solid from 1:10 to 1:40 in ME leads to the TPC from PP (Huang et al., 2017). Another report evaluated polyphenols, showing that the extraction of these compounds from PP was affected by the pH of the solvent, better results were obtained in an acidic environment, and at extra pH above 7.0, lower extraction efficiencies were recorded (Motikar et al., 2021). In the study conducted by Kaderides et al. (2015) in extracting the phenolic compounds of PP by ultrasound, they obtained the highest amount of phenolic compounds in pomegranate equivalent to 13.85 mg of gallic acid in 100 g of PP. In a study on *Pistacia khinjuk*, researchers reported TPC in the extract as 46 mg/g dry weight (Hosseinialhashemi et al., 2021). In a report by Rashid et al. (2022), the TPC of PP was reported to be 217.6 mgGAE/g dry powder. Also, in another study on PP, the amount of TPC was reported as 234.18 mg of GAE/g of extract (Dundar et al., 2023). In another study, phenolic compounds of PP were extracted by ultrasound (at 40°C, 37 Hz). TPC was reported to be 228 mg GAE/100 g of sample (Machado et al., 2019), which is consistent with the results of this study.

### 3.4 Nanoencapsulated compounds particle

The nanoencapsulated compounds’ particle size of the anthocyanin extract of PPP was measured by a Scatroscope1. The average particle size distribution, particle size at peak 1, and its distribution percentages from different extractions are given in Table 3. The average particle size distribution in UE (329 nm) and SEE (422 nm) extracts showed the smallest and largest particles size, respectively. In the SEE, the particle size uniformity was lower. The researchers reported that the mixed emulsion reduced the particle size, which they attributed to the different behavior of biopolymers in reducing the particle size (Tavakoli et al., 2021). In a study on PP, Rashid et al. (2022) stated that the particle size of encapsulated PP (MD/soy protein isolate) was 191.12 nm.

**TABLE 3:** Average particle size distribution, particle size and percentage of distribution in peak 1 in different nanoencapsulated extracts.

Sample	Average particle size distribution (nm)	Percentage of particle distribution in the peak 1 (%)	Particle size at peak 1 (nm)
SEE	422	8.85	95.6
SEWE	344	17.81	112
ME	361	19.62	131
UE	329	29.24	179

Abbreviations: ME, microwave extraction; SEE, solvent ethanol extraction; SEWE, solvent ethanol–water extraction; UE, ultrasound extraction.

### 3.5 FT‐IR

**FIGURE 1:**
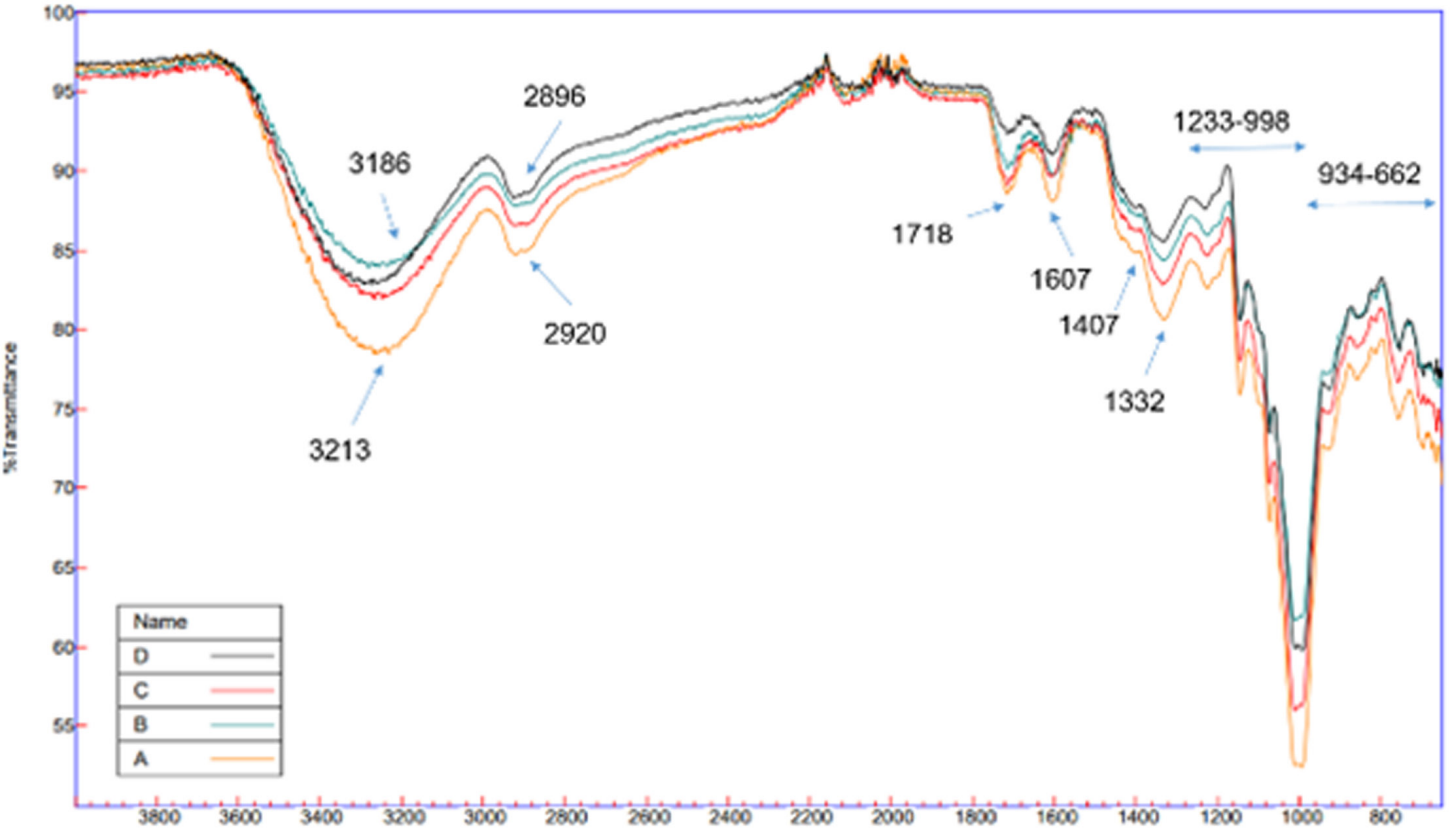
FTIR spectrum for nanoencapsulation samples. A, Microwave extraction; B, Solvent ethanol–water extraction; C, Solvent ethanol extraction; D, Ultrasound extraction.

The FT‐IR spectra for ME extract (A), SEWE extract (B), SEE extract (C), and UE extract (D) are shown in Figure 1. Peaks 3213, 3186, 3334, and 3322 in the samples indicate the O–H bond (Sritham & Gunasekaran, 2017), which indicates the presence of phenolic compounds. Peaks 2920, 2920, and 2896 in ME, SEE, SEWE, and UE indicate C–H in groups –CH3 and –OCH3 (Oliveira et al., 2016). According to the peaks of 3186 and 2920 in the solvent (ethanol/water) and 2896 and 3334 in the ultrasound, it is observed that the amount of hydroxyl and methoxyl compounds in the ultrasound extraction is more and less than the solvent (ethanol/water) sample, respectively. In fact, it can be said that anthocyanidins such as malvidin and petunidin are more abundant in SEWE, while anthocyanidins, such as delphinidin, cyanidin, and pelargonidin are more abundant in SEE. The ethanol solvent leads to the extraction of carbon and hydrogen compounds, whereas the water solvent leads to the extraction of more polar compounds containing hydroxyls. Peak at 1607 in the samples is related to the functional groups of amide, N–H bond and C–O bond in the carboxylic acid (Jamshidi et al., 2020; Oliveira et al., 2016). Peaks at 934 to 662 are related to the C–H bond in aromatic compounds, the amount of which is the same in SEWE extract and UE extract. Phenolic compounds in the ME extract are higher than that in other extracts.

### 3.6 Encapsulation efficiency of anthocyanin

The results Table 4 showed that EEA in SEE extract and SEWE extract had insignificant differences. The ME and UE had the lowest (87.64) and highest (96.15) EEA (%), respectively. In a study by Khazaei et al. (2014) stated that with increasing compounds in the sample, the EE decreased, which is also the case in the study. Also, on encapsulated anthocyanins (core/wall =50%), they reported higher EEA with MD/Arabic gum (89%) than MD/gelatin and MD. The results showed that the EEA with the same core/wall ratio was 96% for UE, 93% for SEE, 92% for SEWE, and 87% for ME. In fact, it shows the superiority of LPSG over Arabic gum for nanoencapsulation of PP anthocyanin compounds. In a report by Robert et al. (2010) on anthocyanin encapsulation, they stated that anthocyanin encapsulation of PP and fruit juice was more efficient than its phenolic compounds with MD and soy protein isolate, which actually indicates that the anthocyanin flavylium cation is bonded with the wall compounds.

**TABLE 4:** Encapsulation efficiency of anthocyanin and phenolic compounds for nanoencapsulated samples.

Sample	EEP (%)	EEA (%)
SEE	75.95 ± 0.63^b^	93.77 ± 0.88^b^
SEWE	86.57 ± 1.19^a^	92.60 ± 1.08^b^
ME	75.96 ± 1.36^b^	87.64 ± 2.45^c^
UE	63.23 ± 2.05^c^	96.15 ± 0.89^a^

*Note*: Different letters in a column indicate significant differences (*p* < .05).

Abbreviations: EEA, encapsulation efficiency of anthocyanin; EEP, encapsulation efficiency of phenol; ME, microwave extraction; SEE, solvent ethanol extraction; SEWE, solvent ethanol–water extraction; UE, ultrasound extraction.

### 3.7 Encapsulation efficiency of phenol (EEP)

The highest EEP (%) was related to the SEWE extract, and the lowest was related to the UE extract, and the amount of EEP (%) of the ME extract and the SEE extract was not significantly different (Table 4). In both extractions, ethanol was used as a solvent, so the polarity of the compounds was similar. The results showed that the phenolic compounds of the SEWE have the highest EEP. Since the chemical structure of the wall and the type of phenolic compounds are suitable, better nanoencapsulation was obtained as a result. The EEP in the UE is at a lower level, which actually indicates inadequate coverage. In a research, Roshanpour et al. (2021) reported EEP using chitosan and *Alyssum homolocarpum* gum at 88.7% (Roshanpour et al., 2021). Also, in another study, researchers stated that EEP with a combined coating of chitosan and locust bean gum was 93.3% (Estakhr et al., 2020). In the research of Rashid et al. (2022), PP extract was encapsulated with MD, soy protein isolate/MD, and reported the EEP of 89.6% and 95.9%, Kaderides et al. (2015) who encapsulated the phenolic compounds extracted with MD, skim milk powder, isolated whey, and Arabic gum reported that the EE of the combination of Arabic gum/MD was 73%, and MD alone was 69% shows the effect of the presence of gum and its emulsifying role in EE. MD is a good protector for the core material. Since MD does not have emulsifying properties, its combination with gum plays an effective role in EE. Koocheki and Hesarinejad (2019) stated that LPSG has the potential to encapsulate nuclear compounds due to its rheological properties, including film formation. The results showed that the combination of LPSG and MD had higher EE for anthocyanin compounds than phenolic compounds due to the difference in electrical charge between anthocyanins (positive charge of anthocyanins) and phenolic compounds.

## 4 CONCLUSION

Due to the importance of anthocyanins and phenolic compounds and also for the optimal use of food industry waste in this study, extraction of anthocyanins and phenolic compounds from PPP was performed with the help of solvent, microwave, and ultrasound. They were then nanoencapsulated with MD and LPSG. The results showed that SEWE had the highest efficiency. However, the ME extract had the highest amount of anthocyanin pigment as well as total phenolic compounds compared with other extraction methods. FT‐IR showed the highest amount of phenolic compounds in the nanoencapsulated extract of ME. In the SEE method, the particle size dispersion was higher. The highest EE of anthocyanins and phenolic compounds were related to UE and SEWE, respectively.

## AUTHOR CONTRIBUTIONS

**Niloofar Zahed:** Formal analysis (equal); methodology (equal); software (equal); visualization (equal); writing – original draft (equal). **Reza Esmaeilzadeh Kenari:** Conceptualization (equal); data curation (equal); investigation (equal); project administration (equal); supervision (equal); writing – review and editing (equal). **Reza Farahmandfar:** Investigation (equal); project administration (equal); resources (equal); supervision (equal); writing – review and editing (equal).

## CONFLICT OF INTEREST STATEMENT

Authors declare no conflict of interest.

## Data Availability

Research data are not shared.

## References

[fsn33362-bib-0001] Abid, M. , Cheikhrouhou, S. , Renard, C. M. , Bureau, S. , Cuvelier, G. , Attia, H. , & Ayadi, M. A. (2017). Characterization of pectins extracted from pomegranate peel and their gelling properties. Food Chemistry, 215, 318–325. 27542481 10.1016/j.foodchem.2016.07.181

[fsn33362-bib-0002] Arabshahi‐Delouee, S. , & Urooj, A. (2007). Antioxidant properties of various solvent extracts of mulberry (*Morus indica* L.) leaves. Food Chemistry, 102(4), 1233–1240.

[fsn33362-bib-0003] Assous, M. T. M. , Abdel‐Hady, M. M. , & Medany, G. M. (2014). Evaluation of red pigment extracted from purple carrots and its utilization as antioxidant and natural food colorants. Annals of Agricultural Sciences, 59(1), 1–7.

[fsn33362-bib-0004] Azmir, J. , Zaidul, I. S. M. , Rahman, M. M. , Sharif, K. M. , Mohamed, A. , Sahena, F. , Jahurul, M. H. A. , Ghafoor, K. , Norulaini, N. A. N. , & Omar, A. K. M. (2013). Techniques for extraction of bioactive compounds from plant materials: A review. Journal of food engineering, 117(4), 426–436.

[fsn33362-bib-0005] Barbosa, M. I. M. J. , Borsarelli, C. D. , & Mercadante, A. Z. (2005). Light stability of spray‐dried bixin encapsulated with different edible polysaccharide preparations. Food Research International, 38(8–9), 989–994.

[fsn33362-bib-0006] Carneiro, H. C. , Tonon, R. V. , Grosso, C. R. , & Hubinger, M. D. (2013). Encapsulation efficiency and oxidative stability of flaxseed oil microencapsulated by spray drying using different combinations of wall materials. Journal of Food Engineering, 115(4), 443–451.

[fsn33362-bib-0007] Chaves, J. O. , De Souza, M. C. , Da Silva, L. C. , Lachos‐Perez, D. , Torres‐Mayanga, P. C. , Machado, A. P. D. F. , Forster‐Carneiro, T. , Vázquez‐Espinosa, M. , González‐de‐Peredo, A. V. , Barbero, G. F. , & Rostagno, M. A. (2020). Extraction of flavonoids from natural sources using modern techniques. Frontiers in Chemistry, 8, 507887. 33102442 10.3389/fchem.2020.507887 PMC7546908

[fsn33362-bib-0008] Chen, L. , Jin, H. , Ding, L. , Zhang, H. , Li, J. , Qu, C. , & Zhang, H. (2008). Dynamic microwave‐assisted extraction of flavonoids from Herba Epimedii. Separation and Purification Technology, 59(1), 50–57.

[fsn33362-bib-0009] Chranioti, C. , Nikoloudaki, A. , & Tzia, C. (2015). Saffron and beetroot extracts encapsulated in maltodextrin, gum Arabic, modified starch and chitosan: Incorporation in a chewing gum system. Carbohydrate Polymers, 127, 252–263. 25965482 10.1016/j.carbpol.2015.03.049

[fsn33362-bib-0010] Duan, W. , Jin, S. , Zhao, G. , & Sun, P. (2015). Microwave‐assisted extraction of anthocyanin from Chinese bayberry and its effects on anthocyanin stability. Food Science and Technology, 35, 524–530.

[fsn33362-bib-0011] Dundar, A. N. , Ozdemir, S. , Uzuner, K. , Parlak, M. E. , Sahin, O. I. , Dagdelen, A. F. , & Saricaoglu, F. T. (2023). Characterization of pomegranate peel extract loaded nanophytosomes and the enhancement of bio‐accessibility and storage stability. Food Chemistry, 398, 133921. 35969988 10.1016/j.foodchem.2022.133921

[fsn33362-bib-0012] Dzah, C. S. (2014). Influence of fruit maturity on antioxidant potential and chilling injury resistance of peach fruit (*Prunus persica*) during cold storage. African Journal of Food, Agriculture, Nutrition and Development, 14(7), 9578–9591.

[fsn33362-bib-0013] Dzah, C. S. , Duan, Y. , Zhang, H. , Wen, C. , Zhang, J. , Chen, G. , & Ma, H. (2020). The effects of ultrasound assisted extraction on yield, antioxidant, anticancer and antimicrobial activity of polyphenol extracts: A review. Food Bioscience, 35, 100547.

[fsn33362-bib-0014] Elsebaie, E. M. , & Essa, R. Y. (2018). Microencapsulation of red onion peel polyphenols fractions by freeze drying technicality and its application in cake. Journal of Food Processing and Preservation, 42(7), e13654.

[fsn33362-bib-0015] Essa, R. Y. , & Mohamed, E. E. (2018). Research article improvement of functional and technological characteristics of spaghetti by the integration of pomegranate peels powder. American Journal of Food Technology, 13, 1–7.

[fsn33362-bib-0016] Estakhr, P. , Tavakoli, J. , Beigmohammadi, F. , Alaei, S. , & Mousavi Khaneghah, A. (2020). Incorporation of the nanoencapsulated polyphenolic extract of *Ferula persica* into soybean oil: Assessment of oil oxidative stability. Food Science & Nutrition, 8(6), 2817–2826. 32566199 10.1002/fsn3.1575 PMC7300055

[fsn33362-bib-0017] Fuleki, T. , & Francis, F. J. (1968). Quantitative methods for anthocyanins. 1. Extraction and determination of total anthocyanin in cranberries. Journal of Food Science, 33(1), 72–77.

[fsn33362-bib-0018] Hosseinialhashemi, M. , Tavakoli, J. , Rafati, A. , & Ahmadi, F. (2021). The application of Pistacia khinjuk extract nanoemulsion in a biopolymeric coating to improve the shelf life extension of sunflower oil. Food Science & Nutrition, 9(2), 920–928. 33598175 10.1002/fsn3.2057 PMC7866579

[fsn33362-bib-0019] Huang, J. , He, W. , Yan, C. , Du, X. , & Shi, X. (2017). Microwave assisted extraction of flavonoids from pomegranate peel and its antioxidant activity. In BIO web of conferences (Vol. 8, p. 03008). EDP Sciences.

[fsn33362-bib-0020] Ilaiyaraja, N. , Likhith, K. R. , Babu, G. S. , & Khanum, F. (2015). Optimisation of extraction of bioactive compounds from *Feronia limonia* (wood apple) fruit using response surface methodology (RSM). Food Chemistry, 173, 348–354. 25466032 10.1016/j.foodchem.2014.10.035

[fsn33362-bib-0021] Jamshidi, M. , Kenari, R. E. , Motamedzadegan, A. , & Biparva, P. (2020). Encapsulation of unsaponifiable matter of rice bran oil bychitosan and *Lepidium perfoliatum* seed gum: Characterization and antioxidant activity. Journal of the American Oil Chemists' Society, 97(10), 1131–1140.

[fsn33362-bib-0022] Kaderides, K. , Goula, A. M. , & Adamopoulos, K. G. (2015). A process for turning pomegranate peels into a valuable food ingredient using ultrasound‐assisted extraction and encapsulation. Innovative Food Science & Emerging Technologies, 31, 204–215.

[fsn33362-bib-0023] Khazaei, K. M. , Jafari, S. M. , Ghorbani, M. , & Kakhki, A. H. (2014). Application of maltodextrin and gum Arabic in microencapsulation of saffron petal's anthocyanins and evaluating their storage stability and color. Carbohydrate Polymers, 105, 57–62. 24708952 10.1016/j.carbpol.2014.01.042

[fsn33362-bib-0024] Kırca, A. , & Cemeroğlu, B. (2003). Degradation kinetics of anthocyanins in blood orange juice and concentrate. Food Chemistry, 81(4), 583–587.

[fsn33362-bib-0025] Koocheki, A. , & Hesarinejad, M. A. (2019). Qodume Shahri (*Lepidium perfoliatum*) seed gum. In Emerging natural hydrocolloids: Emerging natural hydrocolloids (pp. 251–272). Wiley.

[fsn33362-bib-0026] Koocheki, A. , Taherian, A. R. , Razavi, S. M. , & Bostan, A. (2009). Response surface methodology for optimization of extraction yield, viscosity, hue and emulsion stability of mucilage extracted from *Lepidium perfoliatum* seeds. Food Hydrocolloids, 23(8), 2369–2379.

[fsn33362-bib-0027] Kuang, S. S. , Oliveira, J. C. , & Crean, A. M. (2010). Microencapsulation as a tool for incorporating bioactive ingredients into food. Critical Reviews in Food Science and Nutrition, 50(10), 951–968. 21108075 10.1080/10408390903044222

[fsn33362-bib-0028] Kuspradini, H. , Rosiarto, A. M. , Putri, A. S. , & Kusuma, I. W. (2016). Antioxidant and toxicity properties of anthocyanin extracted from red flower of four tropical shrubs. Nusantara Bioscience, 8(2), 135–140.

[fsn33362-bib-0029] Lee, J. , Durst, R. O. B. E. R. T. , & Wrolstad, R. O. N. A. L. D. (2005). AOAC official method 2005.02: Total monomeric anthocyanin pigment content of fruit juices, beverages, natural colorants, and wines by the pH differential method. Official Methods of Analysis of AOAC International, 88(5), 1269–1278. 16385975

[fsn33362-bib-0030] Li, Y. , Guo, C. , Yang, J. , Wei, J. , Xu, J. , & Cheng, S. (2006). Evaluation of antioxidant properties of pomegranate peel extract in comparison with pomegranate pulp extract. Food Chemistry, 96(2), 254–260.

[fsn33362-bib-0031] Machado, A. P. D. F. , Sumere, B. R. , Mekaru, C. , Martinez, J. , Bezerra, R. M. N. , & Rostagno, M. A. (2019). Extraction of polyphenols and antioxidants from pomegranate peel using ultrasound: Influence of temperature, frequency and operation mode. International Journal of Food Science & Technology, 54(9), 2792–2801.

[fsn33362-bib-0032] Malviya, S. , Jha, A. , & Hettiarachchy, N. (2014). Antioxidant and antibacterial potential of pomegranate peel extracts. Journal of Food Science and Technology, 51(12), 4132–4137. 25477693 10.1007/s13197-013-0956-4 PMC4252460

[fsn33362-bib-0033] Maran, J. P. , Sivakumar, V. , Thirugnanasambandham, K. , & Sridhar, R. (2013). Optimization of microwave assisted extraction of pectin from orange peel. Carbohydrate Polymers, 97(2), 703–709. 23911504 10.1016/j.carbpol.2013.05.052

[fsn33362-bib-0034] Masci, A. , Coccia, A. , Lendaro, E. , Mosca, L. , Paolicelli, P. , & Cesa, S. (2016). Evaluation of different extraction methods from pomegranate whole fruit or peels and the antioxidant and antiproliferative activity of the polyphenolic fraction. Food Chemistry, 202, 59–69. 26920266 10.1016/j.foodchem.2016.01.106

[fsn33362-bib-0035] Motikar, P. D. , More, P. R. , & Arya, S. S. (2021). A novel, green environment‐friendly cloud point extraction of polyphenols from pomegranate peels: A comparative assessment with ultrasound and microwave‐assisted extraction. Separation Science and Technology, 56(6), 1014–1025.

[fsn33362-bib-0036] Navas, M. J. , Jiménez‐Moreno, A. M. , Bueno, J. M. , Saez‐Plaza, P. , & Asuero, A. G. (2012). Analysis and antioxidant capacity of anthocyanin pigments. Part IV: Extraction of anthocyanins. Critical Reviews in Analytical Chemistry, 42(4), 313–342.

[fsn33362-bib-0037] Oliveira, R. N. , Mancini, M. C. , Oliveira, F. C. S. D. , Passos, T. M. , Quilty, B. , Thiré, R. M. D. S. M. , & McGuinness, G. B. (2016). FTIR analysis and quantification of phenols and flavonoids of five commercially available plants extracts used in wound healing. Matéria (Rio de Janeiro), 21, 767–779.

[fsn33362-bib-0038] Puértolas, E. , Cregenzán, O. , Luengo, E. , Álvarez, I. , & Raso, J. (2013). Pulsed‐electric‐field‐assisted extraction of anthocyanins from purple‐fleshed potato. Food Chemistry, 136(3–4), 1330–1336. 23194531 10.1016/j.foodchem.2012.09.080

[fsn33362-bib-0039] Rababah, T. M. , Banat, F. , Rababah, A. , Ereifej, K. , & Yang, W. (2010). Optimization of extraction conditions of total phenolics, antioxidant activities, and anthocyanin of oregano, thyme, terebinth, and pomegranate. Journal of Food Science, 75(7), C626–C632. 21535529 10.1111/j.1750-3841.2010.01756.x

[fsn33362-bib-0040] Rana, A. , & Meena, S. (2017). Ultrasonic processing and its use in food industry: A review. International Journal of Chemical Studies, 5(6), 1961–1968.

[fsn33362-bib-0041] Rashid, R. , Masoodi, F. A. , Wani, S. M. , Manzoor, S. , & Gull, A. (2022). Ultrasound assisted extraction of bioactive compounds from pomegranate peel, their nano‐encapsulation and application for improvement in shelf life extension of edible oils. Food Chemistry, 385, 132608. 35279496 10.1016/j.foodchem.2022.132608

[fsn33362-bib-0042] Razavi, S. M. A. , Bostan, A. , Niknia, S. , & Razmkhah, S. (2011). Functional properties of hydrocolloid extracted from selected domestic Iranian seeds. Journal of Food Research, 21(3), 379–389.

[fsn33362-bib-0043] Robert, P. , Gorena, T. , Romero, N. , Sepulveda, E. , Chavez, J. , & Saenz, C. (2010). Encapsulation of polyphenols and anthocyanins from pomegranate (*Punica granatum*) by spray drying. International Journal of Food Science & Technology, 45(7), 1386–1394.

[fsn33362-bib-0044] Roshanpour, S. , Tavakoli, J. , Beigmohammadi, F. , & Alaei, S. (2021). Improving antioxidant effect of phenolic extract of *Mentha piperita* using nanoencapsulation process. Journal of Food Measurement and Characterization, 15(1), 23–32.

[fsn33362-bib-0045] Sadeghian, A. R. , Kadkhodaee, R. , Farhoosh, R. , Koocheki, A. , & Najaf Najafi, M. (2013). Investigating the effect of whey protein‐starch conjugate on quality attributes of oil‐in‐water emulsion. Research and Innovation in Food Science and Technology, 2(2), 139–152.

[fsn33362-bib-0046] Shaban, N. Z. , El‐Kersh, M. A. , El‐Rashidy, F. H. , & Habashy, N. H. (2013). Protective role of *Punica granatum* (pomegranate) peel and seed oil extracts on diethylnitrosamine and phenobarbital‐induced hepatic injury in male rats. Food Chemistry, 141(3), 1587–1596. 23870864 10.1016/j.foodchem.2013.04.134

[fsn33362-bib-0047] Singh, R. P. , Chidambara Murthy, K. N. , & Jayaprakasha, G. K. (2002). Studies on the antioxidant activity of pomegranate (*Punica granatum*) peel and seed extracts using in vitro models. Journal of Agricultural and Food Chemistry, 50(1), 81–86. 11754547 10.1021/jf010865b

[fsn33362-bib-0048] Sritham, E. , & Gunasekaran, S. (2017). FTIR spectroscopic evaluation of sucrose‐maltodextrin‐sodium citrate bioglass. Food Hydrocolloids, 70, 371–382.

[fsn33362-bib-0049] Tavakoli, J. , Abbasi, H. , Zarei Jelyani, A. , & Mousavi Khaneghah, A. (2021). The use of *Salvia macrosiphon* and *Lepidium sativum* Linn. Seed gums in nanoencapsulation processes: Improving antioxidant activity of potato skin extract. Journal of Food Quality, 2021, 1–8.

[fsn33362-bib-0050] Wandrey, C. , Bartkowiak, A. , & Harding, S. E. (2010). Materials for encapsulation. In Encapsulation technologies for active food ingredients and food processing (pp. 31–100). Springer.

